# Supplementary material for: Heterogeneity Challenges in Multiple-Element-Modified Lead-Free Piezoelectric Ceramics
Source: Materials (Basel). 2019 Dec 5;12(24):4049. doi: 10.3390/ma12244049 (PMC6947271; doi:10.3390/ma12244049)
Supplement: Supplementary file 1 [file materials-12-04049-s001.pdf]

materials

Article

Heterogeneity Challenges in Multiple-Element-Modified Lead-Free Piezoelectric Ceramics

Oana Andreea Condurache [1,2,*], Kristian Radan [1], Uroš Prah [1,2], Mojca Otoničar [1,2], Brigita Kmet [1,2], Gregor Kapun [3], Goran Dražić [1,2,3], Barbara Malič [1,2] and Andreja Benčan [1,2]

1. Electronic Ceramics Department, Jožef Stefan Institute, Jamova 39, 1000 Ljubljana, Slovenia; kristian.radan@ijs.si (K.R.); uros.prah@ijs.si (U.P.); mojca.otonicar@ijs.si (M.O.); brigita.kmet@ijs.si (B.K.); goran.drazic@ijs.si (G.D.); barbara.malic@ijs.si (B.M.); andreja.bencan@ijs.si (A.B.)
2. Jožef Stefan International Postgraduate School, Jamova 39, 1000 Ljubljana, Slovenia
3. National Institute of Chemistry, Hajdrihova 19, 1000 Ljubljana, Slovenia; gregor.kapun@ki.si

* Correspondence: oana.condurache@ijs.si

Supplementary Materials S1. Rietveld Refinement for Mn-BC and Mn-AC

In order to determine the phase structure, a Rietveld refinement analysis was performed using Topas R (version 6, Bruker, AXS, Karlsruhe, Germany) software package. The initial structural models for the crystal structure refinement were the structures of KNLNT (orthorhombic, ICSD 195887 and tetragonal, ICSD 195888 crystal systems) [1], modified according to the nominal composition ($0.95(Na_{0.49}K_{0.49}Li_{0.02})(Nb_{0.8}Ta_{0.2})O_3$–$0.05CaZrO_3$) and Mn_3O_4 (tetragonal, ICSD 76088) [2]. The fundamental parameters approach [3] was used to describe the peak profiles, while the background was estimated using a 10th order Chebychev polynomial. The sample displacement, lattice parameters, crystallite size, scale factors, strain, thermal displacement parameters, and atomic coordinates were stepwise refined to obtain a calculated diffraction profile that best fits the experimental pattern. Finally, the quality of the fit was assessed from the goodness-of-fit parameters: R_{wp}, R_p, R_{exp}, R_B, and *G.O.F.*

Table S1. Refined structural parameters for Mn-BC and Mn-BC and fit parameters.

		Mn-BC			Mn-AC	
Phase	KNLNT	KNLNT	Mn_3O_4	KNLNT	KNLNT	Mn_3O_4
Crystal system	Orthorhombic	Tetragonal	Tetragonal	Orthorhombic	Tetragonal	Tetragonal
Space group	*Bmm*2	*P*4*mm*	*I*4$_1$/*amd*	*Bmm*2	*P*4*mm*	I4$_1$/amd
Unit cell parameters	a(Å) = 5.6489(4)	a(Å) = 3.9673(1)	a(Å) = 5.7639(6)	a(Å) = 5.6392(2)	a(Å) = 3.9700(8)	a(Å) = 5.7625(3)
	b(Å) = 3.9730(1)	-	-	b(Å) = 3.9665(1)	-	-
	c(Å) = 5.6253(1)	c(Å) = 3.9959(2)	c(Å) = 9.4566(8)	c(Å) = 5.6246(1)	c(Å) = 3.9928(1)	c(Å) = 9.4605(1)
R_{wp}		9.93			7.98	
R_{exp}		5.95			3.68	
R_p		7.35			6.32	
G.O.F. (χ^2)		1.67			2.17	
R_B	1.859	2.724	7.385	2.539	1.541	3.033

Supplementary Materials S2. Grain Size Distribution of Mn-BC and Mn-AC

The grain size distributions for the samples Mn-BC and Mn-AC are similar. They are both asymmetric distributions, skewed towards larger grains.

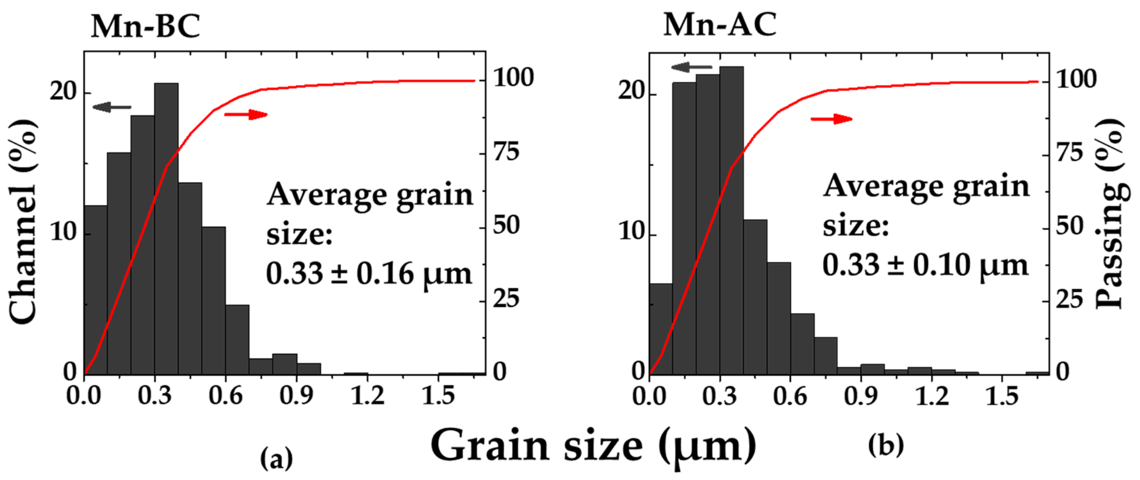

Figure S1. Grain size distribution analysis for (**a**) Mn-BC and (**b**) Mn-AC.

Supplementary Materials S3. Segregation of an Amorphous Phase of Mn-rich Phase at Grain Boundary for Mn-BC Sample

An atomic layer of Mn-rich segregation was found at the grain boundary for the Mn-BC ceramic as the EDXS analysis proved.

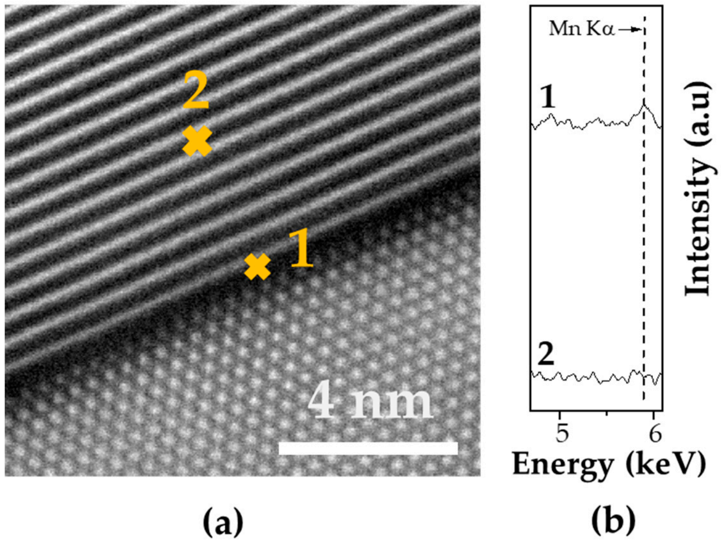

Figure S2. (**a**) ADF STEM image showing a grain boundary in Mn-BC ceramic. (**b**) EDXS analysis showing the Mn Kα peak present at the grain boundary (Spectrum 1) and absent inside the grain interior (Spectrum 2).

References

1. Yin, N.; Jalalian, A.; Zhao, L.; Gai, Z.; Cheng, Z.; Wang, X. Correlation between crystal structures, Raman scattering and piezoelectric properties of lead-free Na0.5K0.5NbO3. *J. Alloys Comp.* **2015**, *652*, 341–345.
2. Boucher, B.; Buhl, R.; Perrin, M. PROPRIETES ET STRUCTURE MAGNETIQUE DE Mn_3O_4, *J. Phys. Chem. Solids* **1970**, *32*, 2429–2437.
3. Cheary, R.W.; Coelho, A. A fundamental parameters approach to X-ray line-profile fitting. *J. Appl. Crystallogr.* **1992**, *25*, 109–121.

© 2019 by the authors. Submitted for possible open access publication under the terms and conditions of the Creative Commons Attribution (CC BY) license (http://creativecommons.org/licenses/by/4.0/).
